# Effect of Vacuum Frying on Changes in Quality Attributes of Jackfruit (*Artocarpus heterophyllus*) Bulb Slices

**DOI:** 10.1155/2014/752047

**Published:** 2014-09-17

**Authors:** Tanushree Maity, A. S. Bawa, P. S. Raju

**Affiliations:** Fruits and Vegetables Technology Discipline, Defence Food Research Laboratory, Siddarthanagar, Mysore, Karnataka 570011, India

## Abstract

The effect of frying temperatures and durations on the quality of vacuum fried jackfruit (JF) chips was evaluated. Moisture content and breaking force of JF chips decreased with increase in frying temperature and time during vacuum frying whereas the oil content increased. The frying time for JF chips was found to be 30, 25, and 20 minutes at 80, 90, and 100°C, respectively. JF chips fried at higher temperature resulted in maximum shrinkage (48%). The lightness in terms of hunter *L* ^*^ value decreased significantly (*P* < 0.05) during frying. Sensory evaluation showed maximum acceptability for JF chips fried at 90°C for 25 min. Frying under vacuum at lower temperatures was found to retain bioactive compounds such as total phenolics, total flavonoids, and total carotenoids in JF chips. Almost 90% of carotenoids were lost from the samples after 30 min of frying at 100°C.

## 1. Introduction

In recent years, fast food snacks industry has emerged as one of the important sectors for the modern consumers with a special desire for fried snack foods. Fried products are liked by all age groups and play an important role in consumer's diet because of their unique flavour and texture. Frying is a quick process which leads to a sterile and dry product with relatively longer shelf life. During the frying process, food is immersed in an oil bath at a temperature above the boiling point of water which results in counter flow of water vapor and oil at the surface of the product [1]. Oil uptake is one of the most important quality parameters of fried food. Oil consumption poses significant health problems such as coronary heart diseases, cancer, diabetes, and hypertension [2] and is irreconcilable with consumers awareness towards the consumption of healthier and low fat food products [3]. Degradation of important nutritional compounds and the generation of toxic molecules in the foodstuff due to high frying temperatures and exposure to oxygen have led to the development of healthy and low fat snack products [4, 5].

Vacuum frying is an excellent alternative to conventional frying which offers significant benefits such as the improvement of fried product safety and quality and reduced oil oxidation because of the low-temperature processing [6]. Vacuum frying is a deep-fat frying process, which is carried out in a closed system, below the atmospheric pressure, substantially reducing the boiling point of water and, hence, the frying temperature. The low frying temperatures and minimal exposure to oxygen are responsible for most of the benefits of the fried products, which include nutrient preservation [7], oil quality protection [8], and reduction in toxic compound generation [9].

Jackfruit is an exotic fruit grown in tropical climates including the Indian subcontinent, southern China, southeastern Asia, middle Africa, and Latin American countries. Consumers like jackfruit for its sweet, fleshy, fibrous, delicious, and attractive golden yellow colored ripe bulbs which is the perianth portion of the fruit. It is rich source of carbohydrates, minerals, dietary fibers, and vitamins such as ascorbic acid and thiamine [10]. However, the edible portion accounts for only 35% of jackfruit making transportation and marketing of the fruit very difficult and uneconomic. Jackfruit contains considerable good amounts of phenolic content and flavonoids which are lost due to various processing techniques including dehydration. The conventional frying of jackfruit is not practicable due to its high sugar content [11]. Higher frying temperature causes charring of the fruit and negligible moisture removal from the fruit. Vacuum frying technique may be suitable for frying sugar rich materials such as jackfruit. Hence the objective of the present study was to study the effect of different frying conditions such as temperature and time on the quality of vacuum fried jackfruit chips.

## 2. Materials and Methods

### 2.1. Sample Preparation

Mature whole jackfruits of firm variety, with an average weight of 8–10 kg, devoid of any visible microbial infection or mechanical fissures were procured from the local fruit market at Mysore, India. Fruits were surface sanitized with chlorinated water (100 ppm). The edible perianth portion (bulbs) was separated manually by slitting open the fruit using stainless steel knives. The bulbs were given a vertical cut to remove the seed. Each pitted bulb was vertically cut into uniform slices (4 × 0.5 × 0.5 cm). The precut bulbs were again subjected to sanitary wash in 30 ppm chlorinated water.

### 2.2. Vacuum Frying

**Figure 1:**
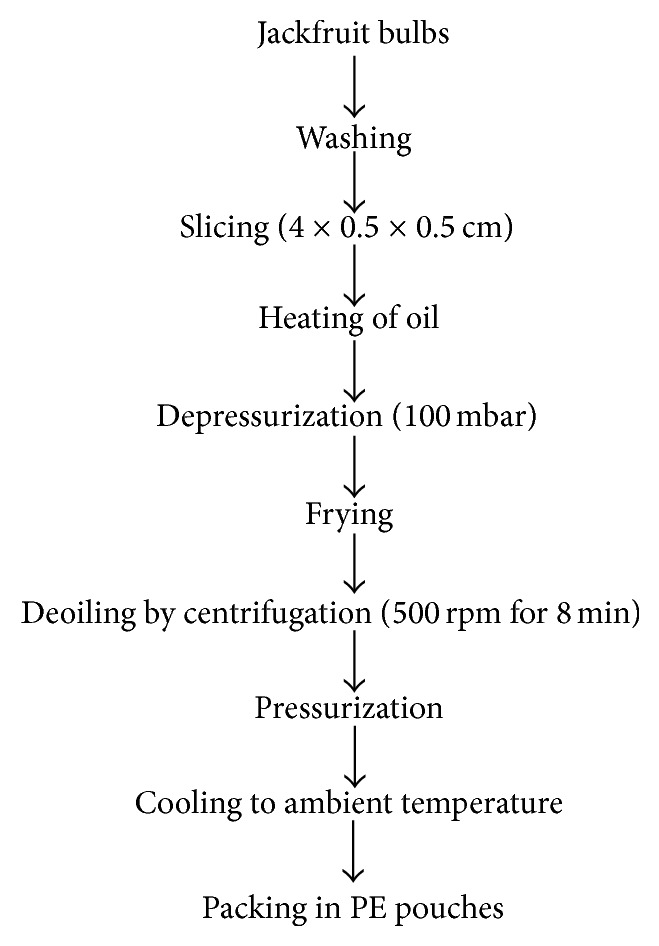
Vacuum frying process of jackfruit chips.

The jackfruit bulb slices were fried in a vacuum fryer equipped with a centrifuge (Vacuum Technologies, Bangalore, India) with a 20 L oil capacity. The working vacuum pressure was 100 mbar for all the experimental conditions. Fresh vegetable oil was used in all the experiments. Once the set temperature of oil is reached, JF slices were loaded in the perforated basket. A batch of 1 Kg JF slices was fried in 20 L of oil. The perforated basket loaded with JF slices was immersed in the hot oil for the prescribed time and is again lifted up from the oil after frying. The JF chips within the basket were centrifuged under vacuum at 500 rpm for 8 minutes to remove the frying oil. Fried JF chips were taken out from the fryer after releasing the vacuum, cooled, and packed in polyethylene pouches (70 *μ*m). The samples were stored at 4°C for further analysis. JF chips were fried at three levels of frying oil temperature (80, 90, and 100°C) for various time intervals (5, 10, 15, 20, 25, and 30 min). The process flow chart is shown in Figure 1.

### 2.3. Moisture Content and Oil Uptake

The moisture content of the fresh JF slices and deoiled JF chips was determined by gravimetric method in triplicates. Moisture content was calculated by weight loss after drying 3 g of coarsely ground sample in a forced convection oven at 105°C to constant weight [12]. Total oil content of coarsely ground JF chips was determined extracting fat with petroleum ether as a solvent by soxhlet extraction apparatus [12]. The determinations were made in triplicate and mean value was reported.

### 2.4. Shrinkage

The vacuum fried JF chips were defatted for 6 h with chloroform before measurement of shrinkage. Shrinkage was calculated from the following, as the difference in volume of the original JF sample and that of the fried JF sample relative to that of the original volume [13]:

(1) Shrinkage  (%)=Vo−VtVo,

where *V*_*o*_ and *V*_*t*_ are the volume of the original JF sample and the volume of the fried sample at time (*t*), respectively. The volume was calculated from (2), using *n*-heptane as the replacement liquid. Consider the following:

(2) Volume=(mph−mp)−(mphs−mp−ms)ρ,

where *m*_ph_ is the mass of the cylindrical tube with *n*-heptane, *m*_p_ is the mass of empty cylindrical tube, *m*_phs_ is the mass of the cylindrical tube with sample and *n*-heptane, *m*_s_ is the mass of the sample, and *ρ* is the density of *n*-heptane.

### 2.5. Color Measurement

The color of the JF chips was measured using a tri-stimulus Colorimeter (Miniscan XE plus, Model number 45/0-S, Hunter Associates Laboratory Inc., Reston, VA, USA) which was calibrated using white and black standard ceramic tiles. The measurements were taken using D-65 illuminant and 10° observer. Color was measured for ten JF chips of each condition and three readings were taken at different locations on the surface of each JF chip for each experimental condition. The color was expressed in terms of *L* ^*^ value [lightness, ranging from zero (black) to 100 (white)], *a* ^*^ value [ranging from +60 (red) to −60 (green)], and *b* ^*^ value [ranging from +60 (yellow) to −60 (blue)].

### 2.6. Texture Analysis

The texture of the fried chips samples was measured using a texture analyzer (TAHdi; Stable Micro Systems, London, UK). A HDP/CFS (crisp fracture support) rig with SS ball probe operated at a test speed of 0.5 mm s^−1^ and over a distance of 5 mm was used to break the sample using a load cell of 5 kg. Pretest and posttest speeds were set at 1 mm s^−1^ and 5 mm s^−1^, respectively. The highest value achieved upon fracturing the sample in the plot was used as the resistance to breakage (Force, *N*). The data obtained from texture profile analysis was used to determine the crispness values. Crispness was expressed as the force at significant break in the first bite.

### 2.7. Sensory Evaluation

Fried jackfruit chips were served to a semitrained panel of twenty members for sensory evaluation in terms of color, crispiness, oiliness, flavor, and overall acceptability using a nine-point hedonic scale for likeness [14]. Panelists were scientific staffs of the laboratory who were trained in the use of attributing rating scale for the characteristics examined. The scores were assigned from extremely liked (9) to disliked extremely (1).

### 2.8. Total Phenolics

Total phenolics (TP) contents of fresh JF slices and defatted JF chips were estimated colorimetrically using Folin-Ciocalteu (FC) reagent [15]. Five grams of fresh JF sample was extracted with 50 mL methanol. The vacuum fried JF chips were defatted by soxhlet extraction for 6 h with chloroform before the methanol extraction [16]. One mL of the extract was mixed with 9 mL of distilled water in a volumetric flask of 25 mL capacity and 1 mL of FC reagent was added and mixture was shaken. After six minutes, 10 mL of sodium carbonate solution (7%) was added and the volume was made up to 25 mL with distilled water. After incubation at room temperature for 90 min, the absorbance of the reaction mixture was measured at 750 nm against a reagent blank using a UV-visible spectrophotometer (Shimadzu-1609, Tokyo, Japan). Quantification was based on the standard curve established with known concentration of gallic acid and the results were expressed as gallic acid equivalents in milligrams per hundred gram dry weight (mg GAE/100 g dw).

### 2.9. Total Flavonoids

Total flavonoids (TF) were determined colorimetrically [17] and expressed as mg catechin equivalents per 100 g on dry weight basis. Sample was extracted in a similar way as discussed in the previous section. One mL of this extract was taken and 4 mL distilled water and 0.3 mL of sodium nitrite solution (5%) were added. After 5 min 0.3 mL of aluminum chloride (10%) was added to the reaction mixture. After 6 min of equilibrium time, 2 mL of sodium hydroxide (1 M) was added. The final volume was made up to 10 mL with distilled water and stirred. The absorbance of the reaction mixture was measured at 510 nm against a prepared reagent blank.

### 2.10. Total Carotenoids

Total carotenoids (TC) in the fried jackfruit chips were determined by extracting 5 g of sample with a solvent mixture containing 40 mL acetone and 60 mL hexane until the JF chips residue turned to colourless [18]. The homogenate was filtered through a filter paper (Whatman number 4) and volume was made up to 50 mL with hexane. Acetone was separated from the mixture by repeated washings with distilled water and 5% sodium chloride solution in a separating funnel. The upper hexane layer with extracted pigment was collected in a volumetric flask, and the volume was made up with hexane. The extraction was carried out under a yellow fluorescent illumination as carotenoids are highly susceptible to light, heat, and air. The absorbance was measured at 450 nm with hexane as blank. The total carotenoids were expressed as *β*-carotene using *A*_1 cm_^1%^ (absorption coefficient) of 2,500 and calculated using the following equation:

(3) Total  carotenoids  (mg100 g) =A450×volume  made  up  (mL)2500×sample  weight  (g)×1000.

### 2.11. Statistical Analysis

The results obtained from both physicochemical analysis and sensory evaluations were subjected to a completely randomized analysis of variance (ANOVA) at *P* < 0.05 and means were separated by Duncan's multiple range tests using Statistica 7 software (Stat Soft, Tulsa, OK, USA).

## 3. Results and Discussion

### 3.1. Moisture

**Figure 2:**
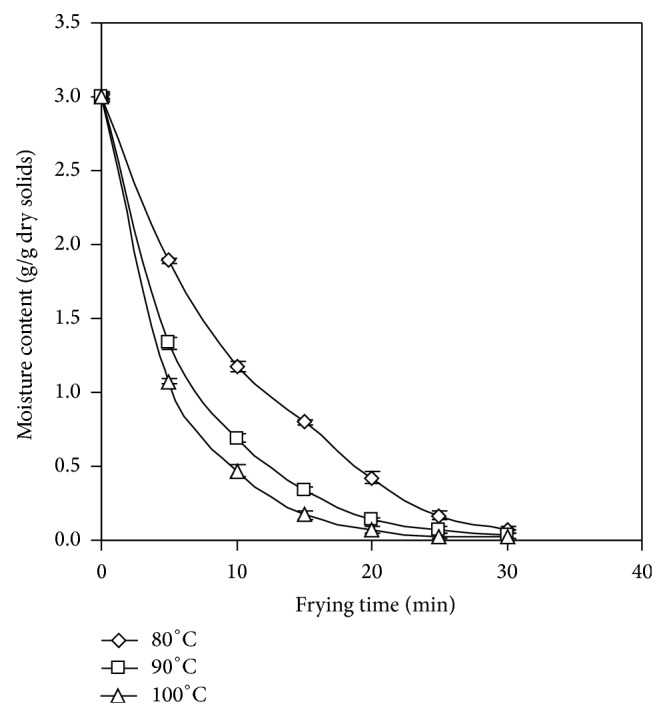
Moisture loss in jackfruit chips during frying.

The moisture loss from JF bulb slices fried under vacuum (100 mbar) at different oil temperatures (80, 90, and 100°C) is shown in Figure 2. The figure shows typical drying curves of JF slices during frying which are similar to the previous studies on vacuum frying of plant originated materials [6, 19]. Since the frying was carried out under vacuum which decreased the boiling point of water, moisture removal was instant from JF slices without much warm-up phase. The phenomenon is in accordance with the findings for vacuum fried potato chips [20]. There were significant (*P* < 0.05) differences in moisture content in the vacuum fried JF chips as effect of frying temperature. The chips fried at 100°C resulted in lower moisture content than the chips fried at 80 and 90°C after the same frying period. Within 5 min of frying, almost 11, 18, and 26% of moisture were removed from JF slices at the frying temperatures of 80, 90, and 100°C, respectively. At 100°C the moisture removal was faster and maximum moisture was removed within 15 min of frying. Moisture removal was gradual but consistent at 80°C. More than 8% moisture was reported to be removed in apple chips within 5 min of vacuum frying at 90°C [21]. At the end of frying (30 min), moisture removal % in JF chips fried at 80, 90, and 100°C was 90, 95, and 97%, respectively.

### 3.2. Oil Content

Oil uptake by the JF chips at different intervals of time and different frying temperatures is presented in Table 1. Results showed that the oil content of JF chips increased with increasing frying temperatures as well as frying time. Oil absorption was rapid during initial 15 minutes of frying at all the frying temperatures. Almost 2.3-, 2.8-, and 3.1-fold higher oil absorption of initial value was recorded by the chips when fried at 80, 90, and 100°C, respectively, which did not change significantly (*P* > 0.05) after 20 and 15 minutes of frying at 90 and 100°C, respectively. After frying for 30 minutes, the oil content was found to be 28.7, 34.79, and 35.15% in JF chips fried at 80, 90, and 100°C, respectively. Absorption of oil was found to be related to the loss of moisture from the chips. This may be due to the diffusion gradient created by the loss of moisture through the surface making the surface dry [21].

### 3.3. Shrinkage

**Figure 3:**
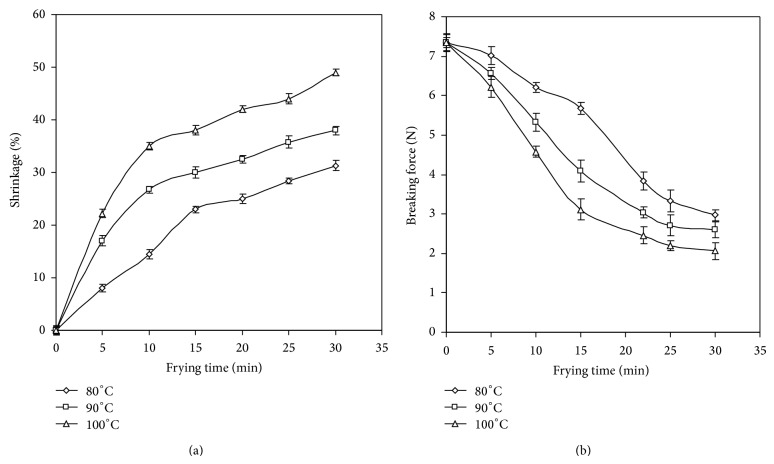
(a) Shrinkage in jackfruit chips during frying; (b) texture of jackfruit chips during frying.

Shrinkage is a common phenomenon during drying.  Figure 3(a) shows the shrinkage in JF chips during frying. As soon as the JF slices were introduced in the hot frying medium, the slices shrunk. Rapid water loss resulted in significant (*P* < 0.05) shrinkage in JF chips during the first 10 min of frying. After 15 min of frying at 90 and 100°C, the change in shrinkage was not significant (*P* > 0.05). This may be due to the fact that at higher temperature sample surface becomes rigid faster due to rapid moisture loss, thus producing increased resistance (case hardening) to volume change [22]. The shrinkage was increased with the progression of frying temperature. At the end of 30 min of frying the % shrinkage in JF chips was 31, 38, and 49% at 80, 90, and 100°C, respectively. High shrinkage was seen in JF chips fried at 100°C which may be due to fast removal of moisture. Similar behavior was also reported in change in shrinkage during vacuum frying of banana chips [23]. Higher shrinkage in potato chips was also reported during high temperature frying [20].

### 3.4. Color

The effect of frying temperature and frying time on the color profile of the JF chips is shown in Table 2. The *L* ^*^ value which indicates the lightness of the fried chips increased during initial 5 min of frying at all the frying temperatures. This may be due to the glossiness acquired by the JF slices due to immersion in frying oil. After that the *L* ^*^ value decreased with increasing frying time. The change in *L* ^*^ value was less at lower frying temperature. Frying for 25 min at 90°C resulted in only 15% reduction in *L* ^*^ value, which continued to reduce to almost 32% in the next 5 min of frying. The change in lightness in JF chips after frying of 30 min at 80°C was almost 16%. However at 100°C, the *L* ^*^ value reduced to almost 50% after 30 min of frying making the chip darker in color. Decrease in *L* ^*^ value has been linked with nonenzymatic browning reactions which accelerates at high temperatures [24, 25]. The *a* ^*^ value of the vacuum fried chips was found to increase with the progress of frying duration at all the frying temperatures. The increase was very rapid at 100°C compared to other frying temperatures. Changes in *a* ^*^ value indicated development of golden brown to dark brown color in JF chips due to browning reactions [6]. The *b* ^*^ value indicates the yellowness of jackfruit bulbs. Frying under vacuum at all the three test temperatures decreased the *b* ^*^ value of the JF chips to different extents. After 15 min of frying the JF chips fried at 100°C had *b* ^*^ values that were significantly lower (*P* < 0.05) than the values corresponding to the JF chips fried at 80 and 90°C. The *b* ^*^ value decreased to almost 11, 18, and 34% at the respective frying temperature of 80, 90, and 100°C after 30 min of frying. The decrease in *b* ^*^ value may be attributed to the instability of carotenoids at higher temperature which was also reported to be interrelated with the decrease in *b* ^*^ value in vacuum fried carrot chips with the degradation of carotenoids at higher frying temperatures [26].

### 3.5. Texture

Texture of a fried product determines its eating quality. Increase in crispiness positively affects the acceptability of product. Crispness in the JF chips was determined as the breaking force of the chips on a texture analyzer. Temperature and time of frying greatly affected the breaking force. In case of fried JF chips, lower values of breaking force indicated higher crispy texture of chips.  Figure 3(b) shows the decrease in breaking force of the chips at all the frying temperatures. High breaking force during initial frying may be due to soft texture of JF slices which in turn was a result of high moisture content [21]. The change in breaking force was slow at 80°C which decreased significantly in the chips fried at 100°C. This may be due to the development of dehydrated crust. After 20 min of frying, there were very less significant changes in the texture values. Further frying would not be necessary for increasing the crispy texture in the product. This would also reduce the cost of the frying process while minimizing nutritional loss. Hence, crispy texture in JF chips was achieved at 30, 25, and 20 minutes at 80, 90, and 100°C, respectively.

### 3.6. Sensory Evaluation

JF chips were assessed for sensory acceptability in terms of color, crispiness, oiliness, flavor, and overall acceptability (Table 3). The sensory score for JF color was found to increase during frying at 80°C. After frying, the yellow color of jackfruit turned into golden-yellow which was rated high on hedonic scale. Frying for longer durations (25–30 min) at 90 and 100°C resulted in lowering of sensory color scores due to surface browning as a result of caramelization. At 100°C, the JF chip achieved golden yellow color in 15 min of frying. Additional frying time resulted in undesirable dark color in JF chips. Crispiness is an important textural attribute which determines the quality of chips [27]. Crispiness in jackfruit slices was found to increase with both temperature and time. Higher temperature of frying achieved crispiness faster as compared to lower frying temperatures. However, sensory scores for crispiness remained unchanged after development of an optimum crispiness. Oiliness of JF chips was found to reduce after 15–20 min of frying which led to concomitant increase in the sensory score for oiliness. During initial frying, the chip was found to be very oily. This may be due to incomplete moisture loss and surface oil absorption. The oiliness decreased with the progression of frying, however, remaining almost unchanged after 20 min till the end of 30 min. The flavor of fresh sample was rated as 9 on hedonic scale. The score was found to decrease during frying at all the frying temperatures. Though the changes in flavor during initial frying periods were not significant, it noticeably changed in chips fried at 100°C. This may be due to degradation of volatile flavor compounds during frying at high temperature. After frying for 30 min the flavor of jackfruit was well retained in the JF chips fried at 80 and 90°C which were lost enormously in JF chips fried at 100°C. Sensory evaluation results suggested that the overall acceptability of JF chips fried at 90°C for 25 min was highest with a score of 8.5. The complete transformation of fresh JF slices into JF chip took almost 15, 25, and 30 min at 100, 90, and 80°C.

### 3.7. Total Phenolics and Total Flavonoids

**Figure 4:**
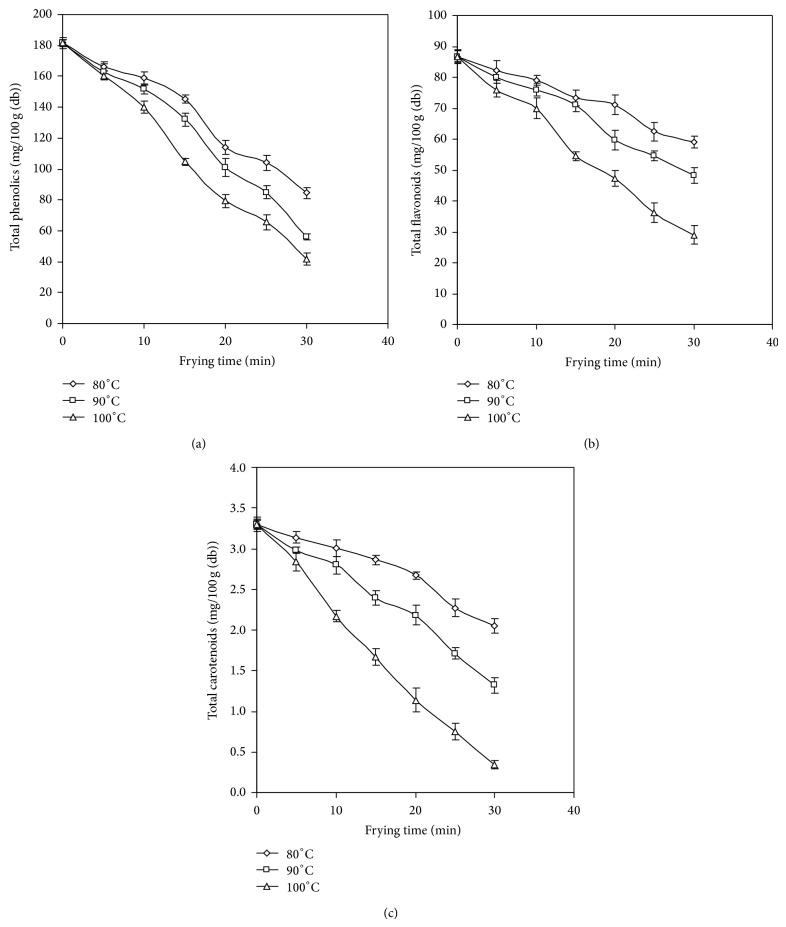
Changes in (a) total phenolics; (b) total flavonoids; and (c) total carotenoids in jackfruit chips during frying.

Phytochemicals such as phenolics and flavonoids play an important role in increasing health benefits [28].  Figure 4(a) represents higher retention of total phenolics (TP) in JF slices fried at 80°C in comparison to the JF chips fried at 90 and 100°C. The JF chips fried at 100°C showed significantly (*P* < 0.05) higher degree of degradation of TP after 10 min of frying. The restricted loss of TP in JF chips after 30 min of frying at 80, 90, and 100°C was found to be 53, 69, and 77%, respectively. This restricted loss could be accredited to application of vacuum during frying which could retain maximum amount of phytochemicals in fried chips such as phenolic compounds by preventing them from oxidation and thermal degradation [29, 30]. Total flavonoids were also found to degrade during frying of JF chips (Figure 4(b)). Frying at 80 and 90°C up to 10 min did not result in significant (*P* < 0.05) loss in flavonoid content of chips. However, flavonoids were found to degrade right from the beginning when chips were fried at 100°C, vis-à-vis from the initial period of 5 minutes to 30 minutes of frying. After frying for 30 minutes almost 32, 45, and 67% of flavonoids were degraded in the JF chips.

### 3.8. Total Carotenoids

The yellow color of JF chips is due to the presence of carotenoids which were found to degrade during frying. Fresh JF slices were found to have 3.03 mg/100 g (dry weight basis) carotenoids. After frying for 5 min 5, 10, and 14% of carotenoids were degraded from initial value in chips fried at 80, 90, and 100°C, respectively. As depicted in Figure 4(c) the degradation was found to be restricted when slices were fried at 80°C. Results also indicated that the rate of degradation of carotenoids was faster after 15 min of frying at all the frying temperatures. Almost 90% carotenoids were degraded when frying was carried out at 100°C for 30 min. At the end of 30 min of frying, the total carotenoids were found to be 2.05, 1.32, and 0.34 mg/100 g in chips processed at 80, 90, and 100°C, respectively. In an earlier work almost 95% loss in total carotenoids during drying of JF slices at 70°C was reported due to oxidation of the pigment, which was accelerated at higher temperature [31]. In our study, the restricted loss of 90% at 100°C may be attributed to the absence of oxygen in vacuum chamber during frying. The carotenoid molecule has a characteristic conjugated polyene which is highly susceptible to degradation due to oxidation [32]. Further, carotenoid is reported to be deteriorated by several researchers during thermal processing depending on the type of raw material and the temperature involved in processing [33, 34].

## 4. Conclusion

Consumers look for products that contribute to their wellness and health; however, even health-conscious consumers are not willing to sacrifice organoleptic properties. Among several deep-fat frying technologies, vacuum frying has a significant strategic importance for future fried food manufacturing. The present study showed that the quality characteristics of fried JF chips such as development of color, texture, and sensory acceptability increased after frying but up to a critical time temperature, while the oil content increased. Lower frying temperatures resulted in maximum retention of bioactive components of the JF chips such as total phenolics, flavonoids, and carotenoids. It may be concluded that JF chips of optimum quality can be obtained from ripened sweet jackfruit slices if the material is fried under vacuum at 90°C for 25 min thus making vacuum fried JF chips an alternative to oily snack foods.

## Figures and Tables

**Table 1:** Oil uptake in jackfruit chips during frying.

Frying time, min	Frying temperature (°C)		
	80	90	100
5	8.04^a^	9.87^a^	10.16^a^
10	12.42^b^	18.54^b^	20.96^b^
15	18.62^c^	27.90^c^	32.02^c^
20	22.14^d^	31.34^cd^	33.15^c^
25	25.4^e^	34.31^d^	34.73^c^
30	28.7^e^	34.79^d^	35.15^c^

Values in same column with different superscripts differ significantly (*P* < 0.05).

**Table 2:** Color values of jackfruit chips at different frying temperatures.

	*L* ^*^ value			*a* ^*^ value			*b* ^*^ value		
	80°C	90°C	100°C	80°C	90°C	100°C	80°C	90°C	100°C
0	61.45^b^	61.45^b^	61.45^ab^	2.31^a^	2.31^a^	2.31^a^	45.52^a^	45.52^a^	45.52^a^
5	71.42^a^	75.29^a^	76.21^a^	3.45^a^	5.85^b^	6.22^b^	45.33^a^	45.18^a^	45.02^a^
10	67.71^a^	70.35^a^	71.36^a^	3.87^b^	6.19^b^	7.07^b^	44.77^a^	44.08^a^	43.54^a^
15	63.34^a^	63.84^b^	60.72^b^	5.62^c^	7.42^b^	9.98^c^	43.65^b^	43.12^b^	40.14^b^
20	60.02^b^	56.81^b^	49.61^c^	6.48^c^	9.32^c^	10.79^c^	42.73^b^	41.23^b^	38.85^b^
25	56.41^c^	52.62^c^	37.36^d^	7.19^d^	9.41^c^	12.13^d^	41.84^b^	39.98^c^	34.61^c^
30	51.65^c^	41.55^d^	31.84^d^	8.32^d^	11.58^d^	14.21^d^	40.34^c^	37.34^c^	30.05^c^

Values with different superscripts in the same column differs significantly (*P* < 0.05).

**Table 3:** Sensory acceptability of jackfruit chips as effect of different frying temperatures.

Time (min.)	Color			Crispiness			Oiliness			Flavor			OAA		
	80°C	90°C	100°C	80°C	90°C	100°C	80°C	90°C	100°C	80°C	90°C	100°C	80°C	90°C	100°C
0	5.00^a^	5.00^a^	5.00^a^	5.22^a^	5.30^a^	5.23^a^	5.50^a^	5.50^a^	5.50^a^	9.00^a^	9.00^a^	9.00^a^	5.00^a^	5.00^a^	5.00^a^
5	5.27^a^	5.28^a^	5.51^a^	5.24^a^	5.80^a^	5.50^a^	5.45^a^	5.61^a^	6.58^b^	8.85^a^	8.78^a^	8.65^a^	5.22^a^	5.81^a^	5.52^a^
10	5.78^b^	5.61^a^	6.00^a^	5.57^b^	6.30^b^	6.36^b^	6.00^a^	7.02^b^	7.35^c^	8.67^a^	8.16^a^	7.88^b^	5.78^b^	6.33^b^	6.13^ab^
15	6.12^b^	6.36^b^	6.71^b^	6.00^b^	7.11^b^	7.85^c^	6.57^b^	7.75^b^	8.23^c^	7.95^b^	7.86^b^	7.05^b^	6.12^b^	7.10^bc^	7.85^b^
20	6.58^b^	6.96^c^	7.42^b^	6.88^b^	7.82^b^	8.51^d^	7.11^b^	7.71^b^	8.25^c^	7.48^b^	7.28^b^	6.76^c^	6.88^b^	7.40^c^	8.42^c^
25	7.16^c^	7.81^c^	6.87^b^	7.25^c^	8.51^c^	8.60^d^	7.00^b^	7.43^b^	8.03^c^	7.27^b^	7.07^b^	6.35^c^	7.25^c^	8.50^d^	8.05^c^
30	7.66^c^	7.38^c^	6.49^b^	7.75^c^	8.60^c^	8.67^d^	6.82^b^	7.35^b^	8.02^c^	7.15^c^	6.62^c^	5.76^d^	7.60^c^	8.12^c^	7.45^b^

Values in same column with different superscripts differ significantly (*P* < 0.05).
